# Prognostic value of body composition on survival outcomes in melanoma patients receiving immunotherapy

**DOI:** 10.3389/fimmu.2023.1261202

**Published:** 2023-11-22

**Authors:** Tianrui Kuang, Lilong Zhang, Zhendong Qiu, Yanbing Zhang, Weixing Wang

**Affiliations:** ^1^ Department of General Surgery, Renmin Hospital of Wuhan University, Wuhan, China; ^2^ Hubei KeyLaboratory of Digestive System Disease, Renmin Hospital of Wuhan University, Wuhan, China

**Keywords:** body composition, immune checkpoint inhibitors, melanoma, skeletal muscle index, skeletal muscle density, visceral fat index, sarcopenia

## Abstract

**Objective:**

The influence of body composition on the effectiveness of immune checkpoint inhibitors (ICIs) in patients with melanoma is still uncertain in clinical practice. Therefore, the objective of this study was to examine the potential association between body composition and clinical outcomes in patients with melanoma undergoing ICIs treatment.

**Methods:**

A systematic literature search was performed across several databases, including PubMed, Embase, Cochrane Library and Google Scholar, to gather relevant studies. The primary outcomes of interest were overall survival (OS) and progression-free survival (PFS), assessed by hazard ratios (HR). Secondary outcomes, such as adverse events (AE), were evaluated using odds ratios (OR).

**Results:**

This meta-analysis comprised ten articles involving a total of 1,283 patients. Systemic analysis of all collected evidence revealed that body composition, including low skeletal muscle index (SMI) (OS: HR = 1.66, 95% CI = 1.13-2.43, p = 0.010; PFS: HR = 1.28, 95% CI = 1.06-1.55, p = 0.009), high subcutaneous adipose tissue density (SMD) (OS: HR = 1.93, 95% CI = 1.09-3.44, p = 0.025; PFS: HR = 1.31, 95% CI = 1.06-1.63, p = 0.012), and sarcopenia (OS: HR = 1.25, 95% CI = 1.03-1.51, p = 0.022; PFS: HR = 1.25, 95% CI = 1.03-1.51, p = 0.022), were significantly associated with OS and PFS in melanoma patients treated with ICIs. However, these markers did not show a significant association with treatment-related adverse events. Interestingly, no significant correlation was found between visceral fat index (VFI) (OS: HR = 0.71, 95% CI = 0.29-1.76, p = 0.462; PFS: HR = 0.98, 95% CI = 0.93-1.02, p = 0.274) and OS or PFS in melanoma patients under ICIs treatment.

**Conclusion:**

Body composition was found to be associated with decreased treatment response and lower long-term efficacy in patients with melanoma undergoing immune checkpoint inhibitor (ICI) therapy. However, it is important to note that body composition did not appear to contribute to increased incidence of adverse events in these patients.

## 1 Introduction

Melanoma, a type of severe skin cancer, is the fifth most prevalent cancer in the US, with more than 91,000 new cases diagnosed in 2018 (1). Although early-stage melanomas (stages I and II) have a 98.4% 5-year survival rate and are usually curable, advanced melanomas (stages III and IV) have a 3-year survival rate as low as 12.2% even with dacarbazine chemotherapy (2, 3). However, immunotherapy using novel immune checkpoint inhibitors (ICIs) has transformed the treatment of advanced melanoma (3). The FDA has approved ipilimumab, pembrolizumab and nivolumab as first-line treatments for advanced melanoma, and clinical trials have demonstrated significantly improved survival rates compared to traditional chemotherapy drugs (4, 5). Notably, the 3-year survival rate associated with ICIs reaching 20.8%, far surpasses that of dacarbazine chemotherapy (6, 7). In summary, ICIs have transformed the landscape of advanced melanoma treatment, probably offering patients prolonged survival and enhanced quality of life.

While ICIs have improved survival rates in cancer patients, the identification of predictive biomarkers for their efficacy remains challenging (8). PD-1/PD-L1 expression shows potential as a biomarker, but its clinical utility is undefined due to variability of analytic methods and inconsistent cut-off values. Therefore, body composition has emerged as a promising alternative (7, 8). Body composition provides a comprehensive assessment of a patient’s physical status by evaluating the volume and quality of muscle and adipose tissue compartments (9, 10). Although body mass index (BMI) is a common indicator of obesity, it has limited ability to assess the clinical prognosis of cancer patients (11). Other markers, such as skeletal muscle index (SMI), subcutaneous fat index (SFI), visceral fat index (VFI) and intermuscular fat index (IFI), have been associated with adverse outcomes in cancer patients, particularly those with sarcopenia—a condition characterized by skeletal muscle loss and dysfunction (12–16). These markers offer a more precise and comprehensive evaluation of body composition and may accurately predict the risks associated with ICIs treatment for melanoma.

Meta-analyses boast several advantages over individual studies as they enable the synthesis of data from multiple datasets, resulting in a pooled effect size and more robust conclusions based on larger patient cohorts. Therefore, the objective of this study was to examine the relationship between pre-immunotherapy body composition and the clinical outcomes of ICIs therapy in patients with melanoma. Specifically, we aimed to investigate the correlation between body composition and tumor treatment response, long-term prognosis and adverse events.

## 2 Materials and methods

### 2.1 Search strategy

This meta-analysis followed the guidelines outlined in the Preferred Reporting Items for Systematic Reviews and Meta-Analyses (PRISMA) statement (17). As of March 2023, systematic literature searches were conducted in PubMed, Embase and Cochrane Library databases. Keywords including “immune checkpoint inhibitors (ICIs)” [Mesh], “skeletal muscle index (SMI),” “visceral fat index (VFI),” “psoas muscle index (PMI),” “skeletal muscle density (SMD),” and “sarcopenia” were searched in the “All Fields” category. We included relevant indicators that are currently representative of body composition. The detailed search strategies are presented in **Table S1**.

### 2.2 Literature inclusion and exclusion criteria

To ensure a robust and comprehensive analysis, specific selection criteria were established. The criteria are as follows: (1) patients who were diagnosed with melanoma, (2) patients receiving ICIs treatment, (3) research on the predictive effect of baseline body composition, and (4) studies that reported at least one of the following conclusions: progression-free survival (PFS), overall survival (OS) and adverse events (AE). Case reports, conference abstracts and comments were excluded to maintain the data quality and consistency. If some studies had overlapping patient cohorts, only those with the most comprehensive and rigorous methodology were chosen, which ensured the selection of high-quality studies and reduced data duplication.

### 2.3 Data extraction

The following information was obtained from the extracted articles: title, first author, publication year, study type, diagnostic methods for sarcopenia or myosteatosis, outcomes assessed, and definitions of these outcomes.

### 2.4 Literature quality evaluation

We used the Newcastle-Ottawa Scale (NOS) for quality assessment of the included studies or cohorts (18). Studies with NOS scores higher than 6 were considered high-quality studies.

### 2.5 Statistical methods and data analysis

HR was employed to measure the association between body composition and clinical outcomes, specifically OS and PFS. To estimate treatment-related adverse events, OR was used. We assessed heterogeneity among the included studies using the chi-square test, with p-value smaller than 0.1 or I^2^ larger than 50% indicating significant heterogeneity (19, 20). In case of significant heterogeneity, a random effects model was applied to account for variations of the study results. Otherwise, a fixed effects model was used. Publication bias was evaluated using Begg’s and Egger’s tests, with *p*-value larger than 0.05 indicating no significant publication bias. To assess results stability, we performed a sensitivity analysis by excluding one study at a time. Statistical significance of the study results was determined at a two-sided p-value smaller than 0.05 (21).

## 3 Results

### 3.1 Literature searching and results screening

**Figure 1:**
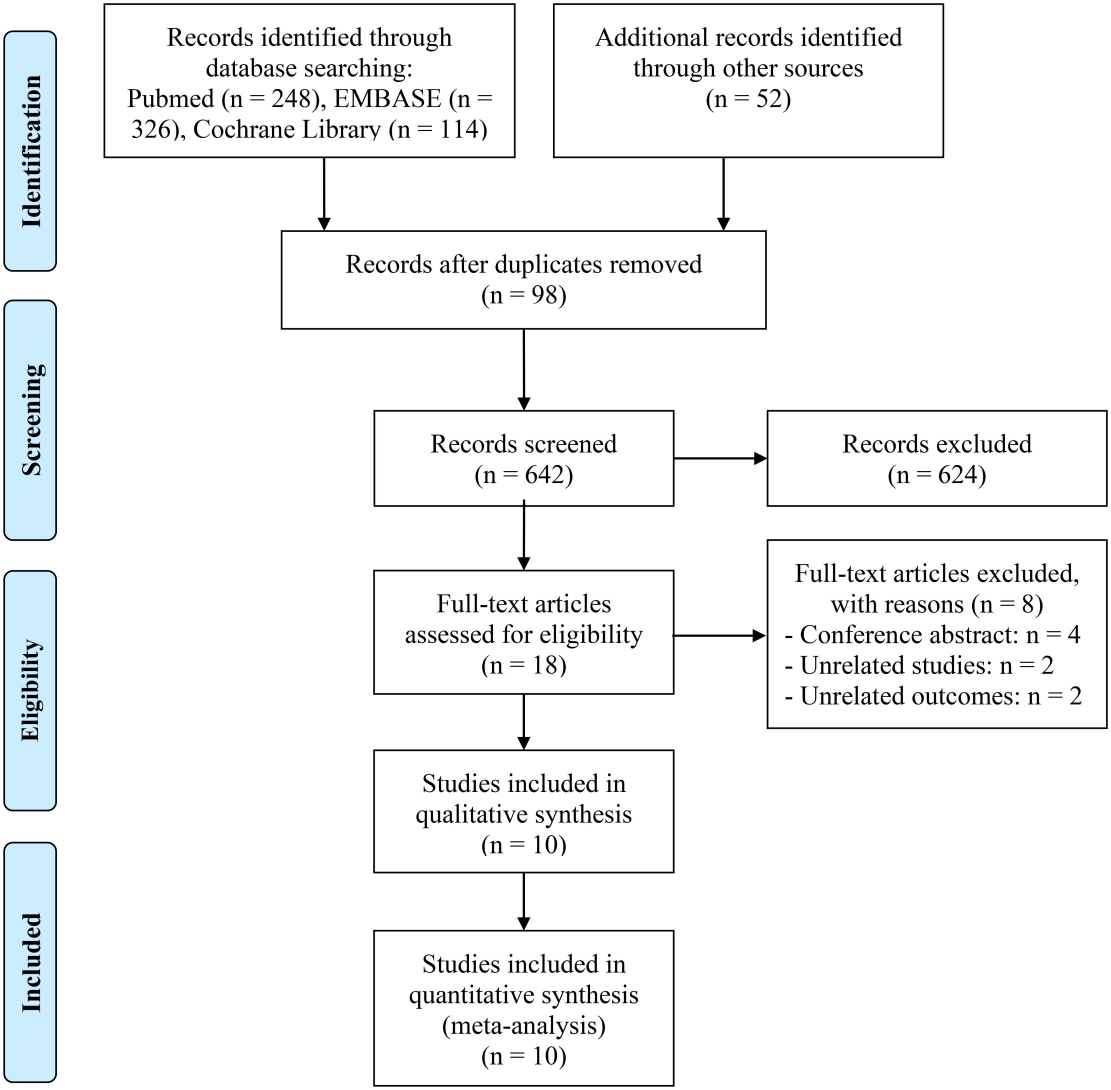
The flow diagram of identifying eligible studies.

**Figure 1** depicts the PRISMA flow diagram that summarizes the selection process. A total of 740 articles were initially retrieved from databases and manual searches, of which 642 remained after duplicates were removed. Following the evaluation of the titles and abstracts, 624 articles were excluded. Full-text reviews were conducted based on predefined inclusion criteria, ultimately resulting in the inclusion of 10 studies with 1294 patients in the final analysis (11, 22–30).

**Table 1** summarizes the key characteristics of the included studies, all of which were designed in a retrospective style and were published after 2020. Sarcopenia was measured using SMI in seven studies, whereas PMI was used to define sarcopenia in one study. Computed tomography (CT) was used as the diagnostic modality for body composition in all articles.

**Table 1:** Main characteristics of the studies included.

Study	Study region	Study period	Study design	Sample size	Age	Male/female	disease status	Treatment	Outcomes	Follow-up (months)	NOS score
Lee et al., 2022 (28)	Korea	06/2015-04/2021	R	266	60 (51-69)^b^	135/131	unresectable or metastatic	Pembrolizumab/nivolumab	SMI, VFI (OS, PFS)	13.9 (6.2-26.1)^c^	8
Faron et al., 2021 (25)	Germany	01/2013-08/2019	R	107	62 ± 15	67/59	metastatic	Pembrolizumab/nivolumab /ipilimumab/nivolumab plus ipilimumab	SMI, VFI (OS)	36^e^	7
Youn et al., 2021 (29)	Canada	2015-2017	R	44	57 (29-79)^a^	25/19	metastatic	Nivolumab	SMD (OS)	–	6
Cortellini et al., 2020 (11)	Italy	04/2015-04/2019	R	27	–	–	advanced	Pembrolizumab/nivolumab/ atezolizumab/others PD-1/PD-L1 agents	SMI (OS, PFS)	20.3^d^	6
Chu et al., 2020 (22)	Canada	2009-2014	R	97	56 (25-91)^a^	58/39	metastatic	Ipilimumab	SMI, SMD (OS, PFS)	–	7
Hu et al., 2020 (27)	USA	01/2014-09/2018	R	156	66 (21-93)^a^	91/65	advanced	Pembrolizumab	PMI (Toxicities)	32.9 (30.4-39.9)^c^	7
Young et al., 2020 (30)	USA	10/2009-10/2018	R	287	63 (20-89)^a^	184/103	metastatic	Anti-PD-1/PD-L1 monotherapy or combination ipilimumab and nivolumab	SMI (OS, PFS, Toxicities), SMD (OS, PFS)	17.3^d^	8
Deike et al., 2019 (24)	Germany	08/2010-04/2017	R	147	60 (49.5-66.5)^b^	62/57	unknown	Ipilimumab	VFI (PFS)	–	7
Heidelberger et al., 2017 (26)	France	08/2014-02/2016	R	68	65 (22-91)^a^	36/32	metastatic	Nivolumab/pembrolizumab	SMI (Toxicities)	6 (0.5-18)^c^	7
Daly et al., 2017 (23)	Ireland	2009-2015	R	84	54 (43-66)^b^	52/32	metastatic	Ipilimumab	SMI (Toxicities)	12.8 (5.5-21.7)^c^	7

^a^amedians with ranges; ^b^median and interquartile range; ^c^medians with 95% confidence interval; ^d^medians; ^e^follow-up period of 3 years; R, retrospective study; SMI, skeletal muscle index; PMI, psoas muscle index; SMD, skeletal muscle density; VFI, visceral fat index; OS, overall survival; PFS, progression-free survival; PD-1, programmed cell death protein 1; PD-L1, programmed cell death 1 ligand 1.

### 3.2 Analysis of the relationship between SMI and ICI efficacy

**Figure 2:**
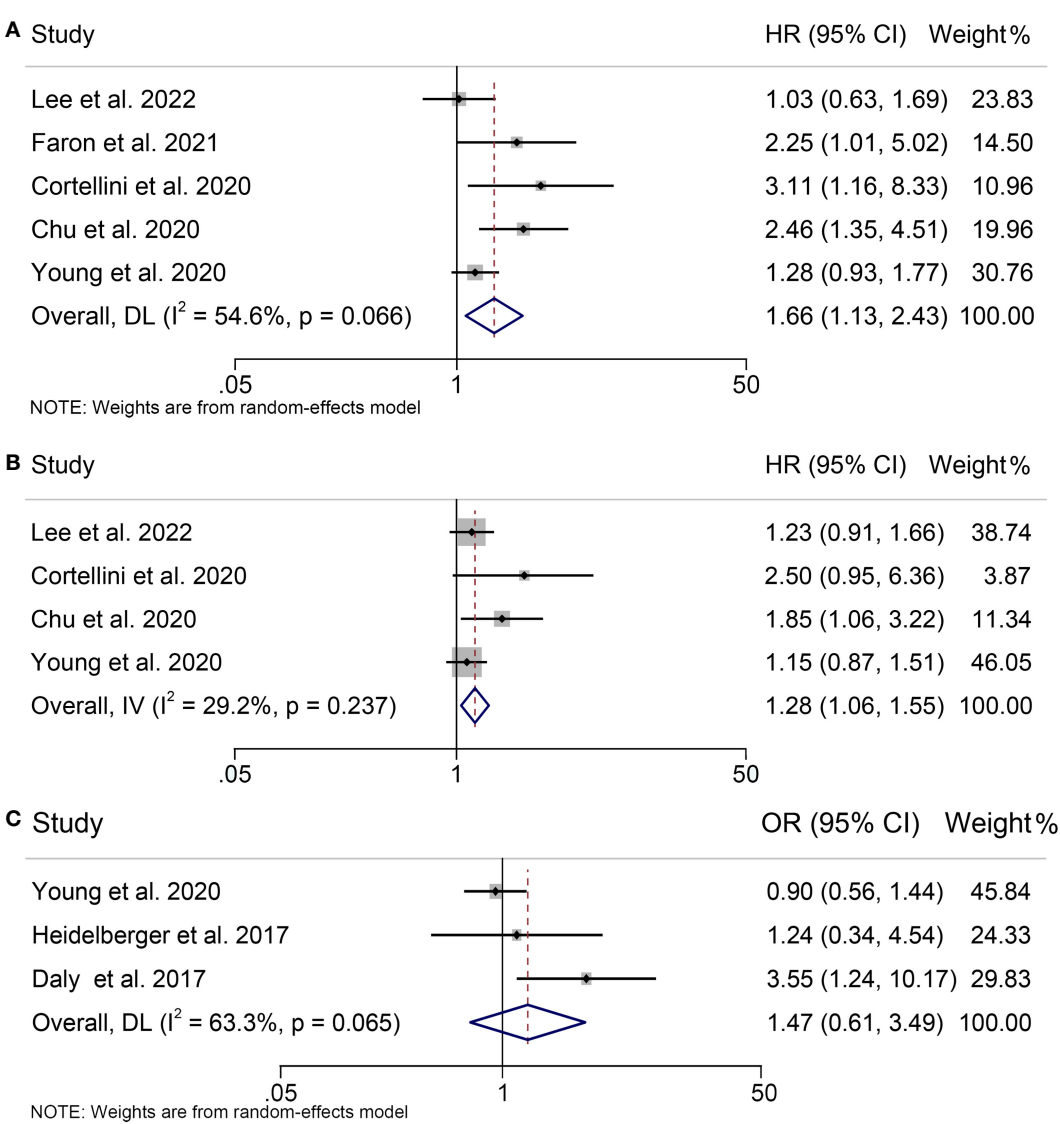
Forest plots demonstrating the relationship between SMI and ICI Efficacy in melanoma patients. **(A)**. SMI and overall survival **(B)**. SMI and progression-free survival **(C)**. SMI and treatment-related toxicity.

Seven studies, concerning 784 melanoma patients, were analyzed to investigate the influence of pretreatment SMI on therapeutic outcomes of ICIs treatment. Our findings showed that patients with low SMI had significantly worse OS than those with high SMI (HR = 1.66, 95% CI = 1.13-2.43, *p* = 0.010; **Figure 2A**). The results of the heterogeneity test were significant (I^2^ = 54.6%, *p* = 0.066), and a random effects model was used to combine the data. Additionally, we evaluated the association between SMI and PFS in three studies with 580 participants. The results revealed a significant association between low SMI and poorer PFS (HR = 1.28, 95% CI = 1.06-1.55, *p* = 0.009; **Figure 2B**), and no significant heterogeneity among those studies (I^2^ = 29.2%, *p* = 0.237), thus a fixed-effects model was applied. The evaluation of the impact of low SMI on treatment-related toxicity showed significant heterogeneity (I^2^ = 63.3%, *p* = 0.065); therefore, a random-effects model was used to combine the data. Our findings indicated that low SMI was not associated with increased risk of treatment-related adverse events (HR = 1.47, 95% CI = 0.61-3.49, *p* = 0.389; **Figure 2C**).

### 3.3 Analysis of the relationship between sarcopenia and ICI efficacy

**Figure 3:**
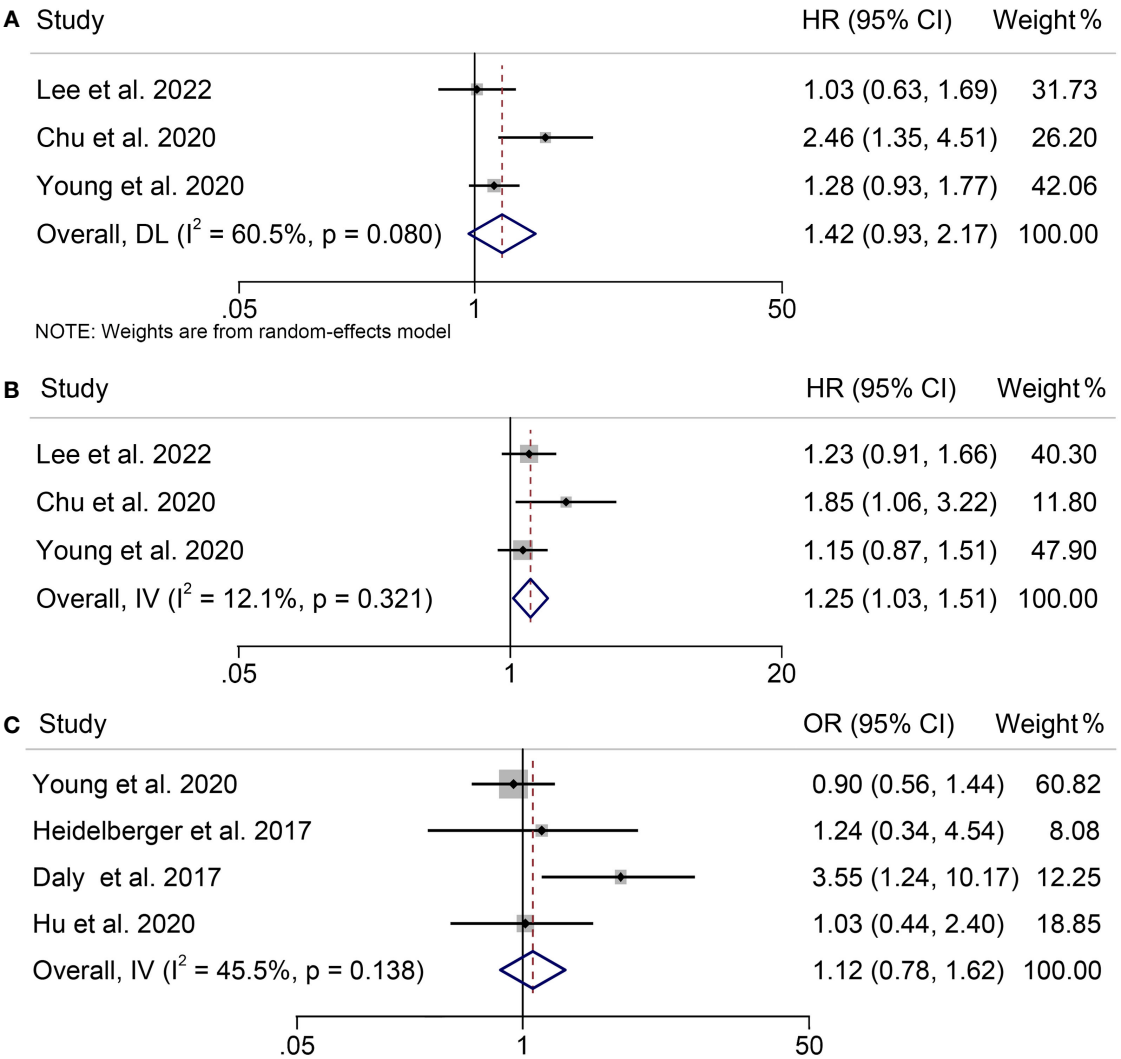
Forest plots demonstrating the relationship between sarcopenia and ICI Efficacy in melanoma patients. **(A)**. sarcopenia and overall survival **(B)**. sarcopenia and progression-free survival **(C)**. sarcopenia and treatment-related toxicity.

A total of 650 patients participated in our meta-analysis, which came from three studies that examined the association between Sarcopenia and OS, PFS and treatment-related adverse events in patients with melanoma under ICIs treatment. According to our findings, the presence of sarcopenia did not have a significant impact on OS in melanoma patients treated with ICIs (HR = 1.42, 95% CI = 0.93-2.17, *p* = 0.108; **Figure 3A**), despite significant heterogeneity among the studies (*p* = 0.080, I^2^ = 60.5%). But sarcopenia was significantly associated with poorer PFS in these patients (HR = 1.25, 95% CI = 1.03-1.51, *p* = 0.022; **Figure 3B**), with no significant heterogeneity (I^2^ = 12.1%, *p* = 0.321); therefore, a fixed-effects model was employed. Additionally, we found no evidence that sarcopenia increased the incidence of treatment-related adverse events in melanoma patients receiving ICIs therapy (HR = 1.12, 95% CI = 0.78-1.62, *p* = 0.544; **Figure 3C**). A fixed-effects model was utilized because of the lack of significant heterogeneity among the studies (I^2^ = 45.5%, *p* = 0.138).

### 3.4 Analysis of the relationship between SMD and ICI efficacy

**Figure 4:**
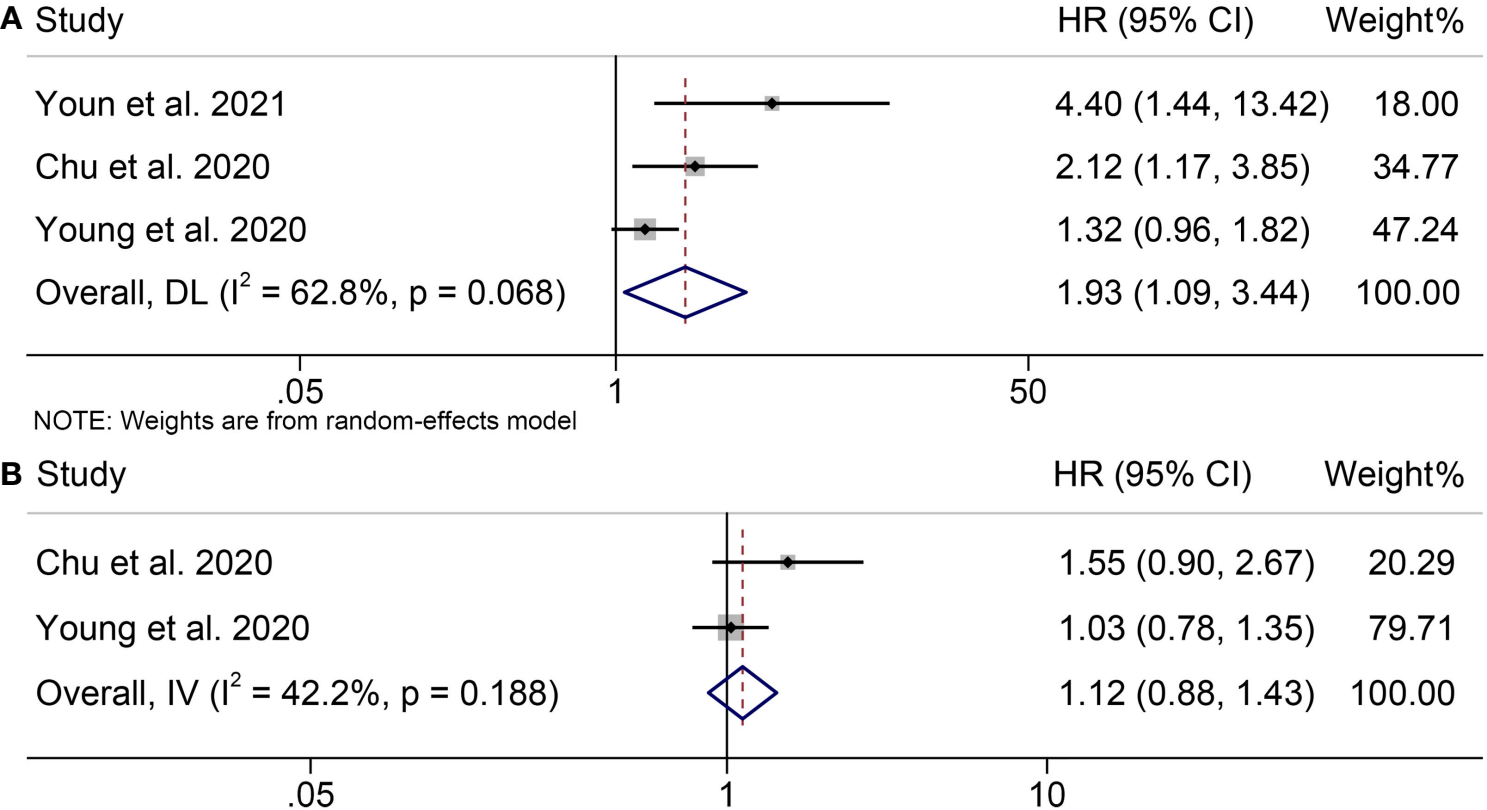
Forest plots demonstrating the relationship between SMD and ICI Efficacy in melanoma patients. **(A)**. SMD and overall survival **(B)**. SMD and progression-free survival.

Three trials including 428 patients examined the association between SMD and OS or PFS in melanoma patients treated with ICIs. Our analysis demonstrated that patients with high SMD had significantly worse OS than those with low SMD (HR = 1.93, 95% CI = 1.09-3.44, *p* = 0.025; **Figure 4A**). Cochran’s Q test and I^2^ statistics revealed significant heterogeneity (I^2^ = 62.8%, *p* = 0.068); therefore, a random-effects model was used for the analysis. The relationship between SMD and PFS in melanoma patients treated with ICIs was investigated. **Figure 4B** illustrated significant heterogeneity among the studies (I^2^ = 57.3%, *p* = 0.104), thus, a random effects model was used. Our results indicated that high SMD was significantly correlated with poorer PFS (HR = 1.31, 95% CI = 1.06-1.63, *p* = 0.012).

### 3.5 Analysis of the relationship between VFI and ICI efficacy

**Figure 5:**
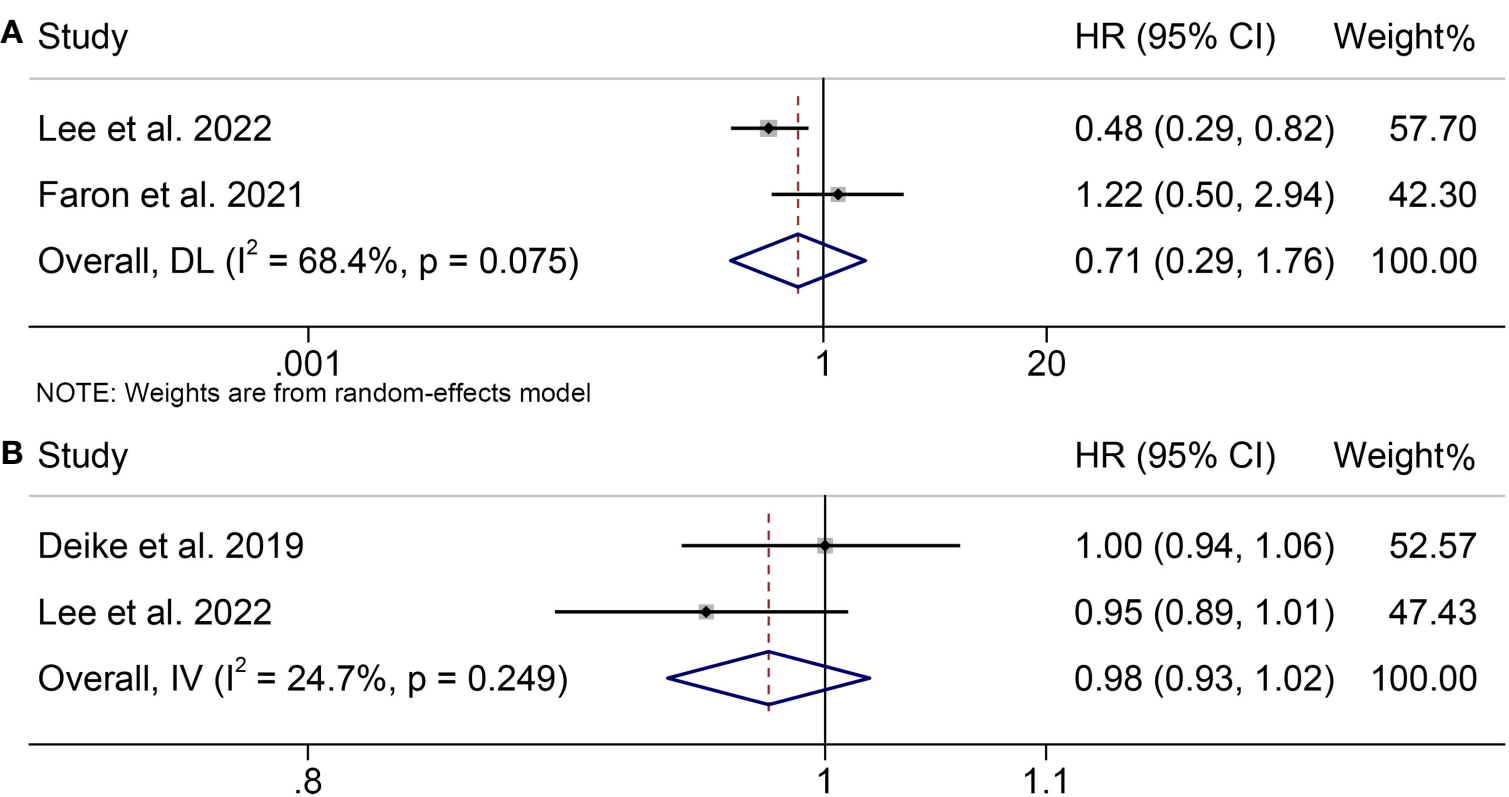
Forest plots demonstrating the relationship between VATI and ICI Efficacy in melanoma patients. **(A)**. VATI and overall survival **(B)**. VATI and progression-free survival.

To assess the impact of pretreatment VFI on OS of patients receiving ICIs, we conducted a meta-analysis of three trials encompassing 784 individuals with melanoma. Our analysis showed no statistical difference, although patients with melanoma with a high VFI had a worse OS compared to patients with melanoma with a low VFI (HR = 0.71, 95% CI = 0.29-1.76, *p* = 0.462; **Figure 5A**). We used a random-effects model because the Cochran’s Q test and I^2^ statistics showed significant heterogeneity among the studies (I^2^ = 68.4%, *p* = 0.075).

Furthermore, we investigated the association between VFI and PFS in melanoma patients treated with ICIs by analyzing two studies involving 384 participants. There was no significant heterogeneity (I^2^ = 24.7%, *p* = 0.249; **Figure 5B**); therefore, a fixed-effects model was used. Our findings indicated that high VFI was not significantly associated with poorer PFS (HR = 0.98, 95% CI = 0.93-1.02, *p* = 0.274).

### 3.6 Sensitivity analysis and publication bias

**Figure 6:**
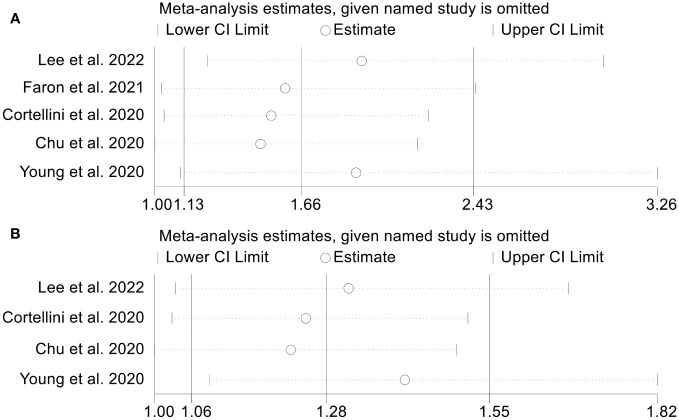
Sensitivity analysis of the association between SMI and ICI Efficacy in melanoma patients. **(A)**. SMI and overall survival **(B)**. SMI and progression-free survival.

As SMI was our primary outcome indicator and was the most frequently included in studies of our analysis, we conducted a test to evaluate publication bias in the relationship between low SMI and both OS and PFS. Begg’s and Egger’s tests were applied, and the results indicated no publication bias in either OS (Egger’s test, *p* = 0.135; Begg’s test, *p* = 0.462) or PFS (Egger’s test, *p* = 0.535; Begg’s test, *p* = 0.755). To ensure the stability of above conclusions, sensitivity analysis was performed by excluding one study at a time, and the results revealed that the combined hazard ratio (HR) for OS remained stable, ranging from 1.520 (95% CI = 1.037-2.229, after excluding Cortellini et al., 2020) to 1.929 (95% CI = 1.232-3.021, after excluding Lee et al., 2022; **Figure 6A**). Similarly, the combined HR for PFS was not significantly altered as well (**Figure 6B**). In a word, our findings are reliable and robust based on the valid research methodology stated above.

## 4 Discussion

This meta-analysis aimed to examine the association between body composition and the efficacy of ICIs in patients with melanoma. Through integrating all available evidence, we discovered significant associations between body composition (including low SMI, high SMD and sarcopenia) and OS and PFS in patients with melanoma undergoing ICIs treatment. However, these indexes did not show significant associations with treatment-related adverse events. Interestingly, no significant correlation was found between VFI and OS or PFS in patients with melanoma treated with ICIs. The reliability and robustness of our findings were confirmed by publication bias and sensitivity analyses. Therefore, our study is highly valuable as it identifies new biomarkers that can assist in predicting patients’ response to ICIs.

Advanced and refractory cancers pose significant challenges to treatment, but ICIs provide potential therapeutic benefits. In order to enhance the clinical effectiveness of ICIs, researchers are actively investigating biomarkers that can facilitate treatment response prediction and guidance on therapy, which is essential for advancing our understanding of cancer immunotherapy and optimizing patient outcomes (31). Although the application of tumor mutational load, microsatellite instability, and PD-L1 immunohistochemical staining may be predictive of favorable therapeutic response, it is hampered by the need for laborious ancillary procedures or painstaking tissue collection (32). In recent years, there has been a growing body of literature discussing how patient body composition-related markers affect the outcome of ICI. Multiple studies have demonstrated a significant impact of body composition on clinical outcomes in various cancer types. For instance, in a separate study involving patients under ipilimumab treatment for metastatic melanoma, sarcopenia was linked to reduced survival rates, while increased infiltration of adipose tissue into skeletal muscle, namely decreased muscle quality, was associated with higher probability of immune-related adverse events (22).

The impact of body composition on the effectiveness of ICIs can be explained by several mechanisms. First, chronic inflammation in cancer often leads to the development of sarcopenia and causes immune dysfunction including T cell exhaustion, which is characterized by reduced effector function, persistent and increased expression of inhibitory receptors, and distinct transcriptional pathways. These factors, such as the activation of pro-inflammatory cytokines and elevated C-reactive protein (CRP) levels, along with a reduction in anti-inflammatory cytokines, can impair anti-tumor immunity and potentially influence the response to ICIs (33–35). Second, there is evidence that the composition of adipose tissue and the immune system can be linked (36). Adipose tissue acts as a reservoir for diverse immune cells, such as macrophages, CD4+ T cells, CD8+ T cells and T regulatory (Treg) cells (37). Mario et al. found that macrophage infiltrates into adipose tissue in obese mice, causing M2 anti-inflammatory macrophage transforming to M1 pro-inflammatory phenotype, whereas this phenomenon is absent in non-obese mice (38, 39). Furthermore, studies have demonstrated that adipose tissue in obese mice has fewer Tregs and effector T cells, as well as an elevated CD8+/CD4+ ratio (40). In addition, Tregs acted as an inhibitor of the inflammatory process in the adipose tissue of lean mice, but in contrast, the number of Tregs was significantly reduced in the adipose tissue of obese mice. Experimental evidence suggests that overweight individuals with pre-existing malignancies may exhibit increased susceptibility to checkpoint inhibition due to a pro-inflammatory state characterized by elevated Th1 responses, macrophage polarization towards M1 pro-inflammatory phenotype, and decreased Treg population in adipose tissue, which indicates that adipose tissue in overweight individuals generates an inflammatory microenvironment that may potentially influence the effectiveness of ICIs and impact the immune response against malignancies (41). However, this hypothesis needs to be tested with more clinical data and further experiments. Therefore, it is crucial to consider the potential negative impact of body composition on ICI efficacy in clinical practice. Further research is needed to determine whether body composition could enhance the therapeutic benefits of ICIs immunotherapy and to elucidate the underlying mechanisms.

Our study had several limitations that should be acknowledged. Firstly, the majority of included studies were retrospective, and their cohort volumes were relatively small. Secondly, the underlying selection bias in original studies may have contributed to the observed heterogeneity. As a meta-analysis relying on previously published articles, our study was subject to inherent limitations specific to this article genre. Thirdly, due to insufficient data, important clinical features known to impact ICI efficacy, such as age and sex, were not taken into account. Lastly, body composition remains a controversial biomarker in clinical practice, and the standardization of its assessment are still challenging. Further studies are required to elucidate and establish the optimal criterion for body composition.

## 5 Conclusions

In this study, the results indicated that body composition could negatively affect both OS and PFS in cancer patients treated with ICIs, as determined by univariate and adjusted HR. Specifically, low SMI could serve as a valuable predictor of ICI treatment response and should be assessed prior to initiating ICI therapy.

## Data availability statement

The original contributions presented in the study are included in the article/**Supplementary Material**. Further inquiries can be directed to the corresponding authors.

## Author contributions

TK: Writing – original draft. LZ: Investigation, Writing – original draft. ZQ: Formal analysis, Software, Writing – review & editing. YZ: Writing – review & editing. WW: Writing – review & editing.
