# Supplementary material for: Prognostic value of body composition on survival outcomes in melanoma patients receiving immunotherapy
Source: Front Immunol. 2023 Nov 22;14:1261202. doi: 10.3389/fimmu.2023.1261202 (PMC10704136; doi:10.3389/fimmu.2023.1261202)
Supplement: Supplementary file 1 [file Table_1.docx]

**Table S1** Detailed search strategy

((((((((((((((((((((((((((((skeletal muscle index) OR (SMI)) OR (psoas muscle index)) OR (PMI)) OR (subcutaneous adipose index)) OR (SAI)) OR (subcutaneous fat index)) OR (SFI)) OR (visceral adipose index)) OR (VAI)) OR (visceral fat index)) OR (VFI)) OR (intramuscular adipose index)) OR (IMAI)) OR (intramuscular fat index)) OR (IMFI))) ) OR (muscle surface area)) OR (MSA)) OR (skeletal muscle density)) OR (SMD)) OR (myosteatosis))) OR (((((((((((((((((((((((((sarcopenia) OR (sarcopenic)) OR (muscle index)) OR (muscle mass)) OR (muscle depletion)) OR (muscular atrophy)) OR (muscle strength)) OR (muscle quality)) OR (muscle quantity)) OR (muscle loss)) OR (Myopenia)) OR (Muscle atrophy)) OR (Muscle wasting)) OR (Muscle attenuation)) OR (Muscle deletion)) OR (Muscle weak)) OR (Muscle reduction)) OR (Muscular wasting)) OR (Muscular attenuation)) OR (Muscular deletion)) OR (Muscular loss)) OR (Muscular weak)) OR (Muscular reduction)) OR (Muscular strength)) OR (Muscular mass)))))) AND (((anti-CTLA4 antibody) OR (anti-CTLA4 antibodies)) OR (((((((((((((((((((((((((((((((((((((((((((((((((((((((Immune Checkpoint Inhibitors) OR (Checkpoint Inhibitors, Immune)) OR (Immune Checkpoint Inhibitor)) OR (Checkpoint Inhibitor, Immune)) OR (Immune Checkpoint Blockers)) OR (Checkpoint Blockers, Immune)) OR (Immune Checkpoint Blockade)) OR (Checkpoint Blockade, Immune)) OR (Immune Checkpoint Inhibition)) OR (Checkpoint Inhibition, Immune)) OR (PD-L1 Inhibitors)) OR (PD L1 Inhibitors)) OR (PD-L1 Inhibitor)) OR (PD L1 Inhibitor)) OR (Programmed Death-Ligand 1 Inhibitors)) OR (Programmed Death Ligand 1 Inhibitors)) OR (PD-1-PD-L1 Blockade)) OR (Blockade, PD-1-PD-L1)) OR (PD 1 PD L1 Blockade)) OR (CTLA-4 Inhibitors)) OR (CTLA 4 Inhibitors)) OR (CTLA-4 Inhibitor)) OR (CTLA 4 Inhibitor)) OR (Cytotoxic T-Lymphocyte-Associated Protein 4 Inhibitors)) OR (Cytotoxic T Lymphocyte Associated Protein 4 Inhibitors)) OR (Cytotoxic T-Lymphocyte-Associated Protein 4 Inhibitor)) OR (Cytotoxic T Lymphocyte Associated Protein 4 Inhibitor)) OR (PD-1 Inhibitors)) OR (PD-1 Inhibitor)) OR (PD 1 Inhibitors)) OR (Inhibitor, PD-1)) OR (PD 1 Inhibitor)) OR (Programmed Cell Death Protein 1 Inhibitor)) OR (Programmed Cell Death Protein 1 Inhibitors)) OR ("Immune Checkpoint Inhibitors"[Mesh])))))))) OR (pembrolizumab)) OR (nivolumab)) OR (atezolizumab)) OR (ipilimumab)) OR (avelumab)) OR (tremelimumab)) OR (durvalumab)) OR (cemiplimab))) OR (anti-PD-1 antibodies)) OR (anti-PD-1 antibody))) OR (anti-PD-L1 antibody)) OR (anti-PD-L1 antibodies)))
